# O Strain Atrial Esquerdo é a Parte que Falta para Diagnosticar a Disfunção Diastólica em Crianças com Doença Renal Crônica?

**DOI:** 10.36660/abc.20240257

**Published:** 2024-06-25

**Affiliations:** 1 Faculdade de Ciências Médicas de Minas Gerais Belo Horizonte MG Brasil Faculdade de Ciências Médicas de Minas Gerais – Pós-Graduação, Belo Horizonte, MG – Brasil; 2 Hospital Felício Rocho Belo Horizonte MG Brasil Hospital Felício Rocho – Ecocardiografia, Belo Horizonte, MG – Brasil

**Keywords:** Átrios do Coração, Rim, Ecocardiografia/métodos, Criança

A ecocardiografia é a modalidade de imagem mais importante para avaliar a função diastólica, parte essencial da avaliação da função cardíaca.^1^

Desde a sua publicação em 2016, as recomendações da ASE/EACVI para avaliação da função diastólica foram validadas em diferentes coortes, mas apresentam resultados conflitantes, principalmente quando se consideram pacientes com fração de ejeção (FE) preservada. Quando pacientes com FE preservada foram estudados em subgrupo separado, observou-se pior sensibilidade.^2^

Esse grupo específico exige índices alternativos, que poderiam ser incorporados ao algoritmo para diagnosticar com precisão a ICFEP.^3^

Em crianças, a avaliação da função diastólica utilizando estes parâmetros habituais permanece mal definida no diagnóstico e classificação da gravidade da disfunção diastólica. Por exemplo, a reversão das ondas E/A não é frequente em crianças com disfunção diastólica estabelecida.^4,5^

Um método não invasivo de medição da rigidez do átrio esquerdo (AE), onde E/e’ foi usado como substituto da pressão capilar pulmonar e corrigindo-a com *strain* do AE, parece melhorar a precisão da avaliação das pressões de enchimento do ventrículo esquerdo (VE) em crianças.

Da mesma forma, o índice de enchimento do AE, calculado como a razão entre a onda E mitral e o *strain* do reservatório do AE, poderia diagnosticar disfunção diastólica com pressão de enchimento do VE elevado melhor do que E/e’.^6^

Existem fortes evidências em adultos de que o volume e a função do AE são potentes preditores de diferentes desfechos cardíacos. A maioria dos pesquisadores utiliza cada vez mais esses dados para avaliar a função cardíaca em vários cenários de doenças cardiovasculares. O estudo do AE que avalia a função diastólica do VE é uma das aplicações mais significativas.^7^

Na população pediátrica, os estudos ecocardiográficos sobre a fisiologia e função do AE são escassos.

O *strain* do átrio esquerdo tornou-se um parâmetro valioso para avaliar a função diastólica do ventrículo esquerdo e estimar as pressões de enchimento do VE. O *strain* do AE foi inicialmente medido por Doppler tecidual. Avanços na ecocardiografia com *speckle tracking* aumentaram a quantificação do pico de expansão do AE durante a sístole do VE, denominada *strain* do reservatório do AE.^8^ O *strain* do reservatório do AE está intimamente correlacionado com miopatia, fibrose do AE e pressões de enchimento e pode prever insuficiência cardíaca, acidente vascular cerebral e mortalidade taxas na população geral.^9^ Poucos estudos relataram o desempenho do conduto do AE ou do *strain* da bomba na função diastólica do VE.

A *Task Force* recomenda medir o AE por meio do *speckle tracking* em cortes apicais de 4 e 2 câmaras.^10^ Na prática diária, a obtenção de imagens do teto do AE pode ser desafiadora e, em alguns casos, há uma grande variação entre o *strain* do AE no septo interatrial e a parede livre da AE. Um estudo mostrou que o *strain* do conduto do AE estava diretamente relacionado à velocidade diastólica precoce do anel mitral (e’) e uma relação inversa com a fração do volume extracelular miocárdico medida por ressonância magnética cardíaca.^11^

Foi demonstrado que a redução no *strain* do reservatório do AE precede alterações nos volumes do AE, pode estimar as pressões de enchimento do VE em alguns casos e pode ser uma ferramenta robusta para detectar disfunção diastólica subclínica e prever o desenvolvimento de insuficiência cardíaca congestiva em pacientes com FEVE normal.^12,13^

O estudo de Penachio et al.,^14^ cobrindo pacientes pediátricos com doença renal crônica (DRC) é muito apropriado porque a disfunção diastólica do VE é frequentemente encontrada nesta população específica e está frequentemente associada a complicações cardiovasculares.

Por outro lado, existem limitações para estabelecê-la, sendo também um desafio quantificar a disfunção diastólica em crianças.

O grupo comprovou que a obtenção do *strain* do AE em aparelho ecocardiográfico comercial é uma técnica viável na avaliação diastólica na prática diária, com variabilidade intra e interobservador encorajadora. Eles encontraram dados interessantes confirmando que a média isolada de E/e’, embora mais alta em crianças com DRC do que em controles, estava acima dos limites normais em apenas um paciente. Por outro lado, a rigidez e o índice de enchimento do AE foram maiores. O *strain* do reservatório do AE foi menor na hipertrofia do VE e na hipertensão não controlada.

Parece adequado incluir uma avaliação rotineira do *strain* do AE nesta população pediátrica específica para obter uma avaliação mais abrangente e integrativa.
