# Supplementary material for: O Strain Atrial Esquerdo é a Parte que Falta para Diagnosticar a Disfunção Diastólica em Crianças com Doença Renal Crônica?
Source: Arq Bras Cardiol. 2024 Jun 25;121(3):e20240257. [Article in Portuguese] doi: 10.36660/abc.20240257 (PMC11216338; doi:10.36660/abc.20240257)
Supplement: Translation

# Translation: Is Left Atrial Strain the Missing Part to Diagnose Diastolic Dysfunction in Children with Chronic Kidney Disease?

**DOI:** 10.36660/abc.20240257i

**Affiliations:** 1 Belo Horizonte MG Brazil Faculdade de Ciências Médicas de Minas Gerais – Pós-Graduação, Belo Horizonte, MG – Brazil; 2 Belo Horizonte MG Brazil Hospital Felício Rocho – Ecocardiografia, Belo Horizonte, MG – Brazil

**Keywords:** Heart Atria, Kidney, Echocardiography/methods, Child

Echocardiography is the most critical imaging modality for assessing diastolic function, an essential part of the cardiac function evaluation.^1^

Since its publication in 2016, the ASE/EACVI recommendations for evaluating diastolic function have been validated in different cohorts but have shown conflicting results, especially when considering patients with preserved ejection fraction (EF). When patients with preserved EF were studied in a separate subgroup, a poorer sensitivity was observed.^2^

This specific group calls for alternative indices, which could be incorporated into the algorithm to diagnose HFpEF accurately.^3^

In children, the assessment of diastolic function using these usual parameters remains poorly defined in diagnosing and grading the severity of diastolic dysfunction. For example, E/A wave reversal is not frequent in children with established diastolic dysfunction.^4,5^

A noninvasive method of measuring left atrial (LA) stiffness, where E/e’ was used as a substitute for pulmonary capillary wedge pressure and correcting it with LA strain, seems to improve the accuracy of assessment of left ventricle (LV) filling pressures in children.

In the same way, the LA filling index, calculated as the ratio of mitral E wave to LA reservoir strain, could diagnose diastolic dysfunction with elevated LV filling pressure better than E/e’.^6^

Strong evidence exists in adults that LA volume and function are potent predictors of different cardiac outcomes. Most researchers increasingly use that data to evaluate cardiac function in various cardiovascular disease scenarios. LA study assessing LV diastolic function is one of the most significant applications.^7^

In the pediatric population, echocardiographic studies on LA physiology and function are scarce.

Left atrium strain has become a valuable parameter for assessing left ventricle diastolic function and estimating LV filling pressures. LA strain was initially measured by tissue Doppler. Advancements in speckle tracking echocardiography have incremented the quantification of the peak expansion of the LA during LV systole, named LA reservoir strain.^8^ LA reservoir strain closely correlates with myopathy, LA fibrosis, and filling pressures and can predict heart failure, stroke, and mortality rates in the general population.^9^ Few studies have reported on the performance of LA conduit or pump strain in LV diastolic function.

The Task Force recommends measuring LA by speckle tracking in apical 4-and 2-chamber views.^10^ In daily practice, imaging the LA roof can be challenging, and, in some cases, there is wide variation between LA strain in the interatrial septum and LA-free wall. One study showed that LA conduit strain was directly related to early diastolic mitral annulus velocity (e’) and an inverse relation with myocardial extracellular volume fraction measured by cardiac magnetic resonance.^11^

It has been demonstrated that reduction in LA reservoir strain precedes changes in LA volumes, can estimate LV filling pressures in some cases, and can be a robust tool to detect subclinical diastolic dysfunction and predict the development of congestive heart failure in patients with normal LVEF.^12,13^

The study by Penachio et al.^14^ covering pediatric patients with chronic kidney disease (CKD) is very reasonable because LV diastolic dysfunction is frequently found in this particular population and is often associated with cardiovascular complications.

On the other hand, there are limitations to establishing it, and it is also a challenge to quantify diastolic dysfunction in children.

The group proved that obtaining LA strain from a commercial echocardiography machine is a feasible technique concerning diastolic evaluation in daily practice, with encouraging intra-and interobserver variability. They found exciting data confirming that isolated average E/e’, although higher in CKD children than controls, was above normal limits in only one patient. On the other hand, LA stiffness and filling index were higher. LA reservoir strain was lower in LV hypertrophy and uncontrolled hypertension.

It seems adequate to include a routine evaluation of LA strain in this specific pediatric population to obtain a more comprehensive and integrative appraisal.
